# Arf6 Can Trigger Wave Regulatory Complex-Dependent Actin Assembly Independent of Arno

**DOI:** 10.3390/ijms21072457

**Published:** 2020-04-02

**Authors:** Vikash Singh, Anthony C. Davidson, Peter J. Hume, Vassilis Koronakis

**Affiliations:** Department of Pathology, Tennis Court Road University of Cambridge, Cambridge CB2 1QP, UK; Vs399@cam.ac.uk (V.S.); acd49@cam.ac.uk (A.C.D.); pjh53@cam.ac.uk (P.J.H.)

**Keywords:** Arf GTPases, actin cytoskeleton, wave regulatory complex, phagocytosis, macrophages, host–pathogen interplay

## Abstract

The small GTPase ADP-ribosylation factor 6 (Arf6) anchors at the plasma membrane to orchestrate key functions, such as membrane trafficking and regulating cortical actin cytoskeleton rearrangement. A number of studies have identified key players that interact with Arf6 to regulate actin dynamics in diverse cell processes, yet it is still unknown whether Arf6 can directly signal to the wave regulatory complex to mediate actin assembly. By reconstituting actin dynamics on supported lipid bilayers, we found that Arf6 in co-ordination with Rac1(Ras-related C3 botulinum toxin substrate 1) can directly trigger actin polymerization by recruiting wave regulatory complex components. Interestingly, we demonstrated that Arf6 triggers actin assembly at the membrane directly without recruiting the Arf guanine nucleotide exchange factor (GEF) ARNO (ARF nucleotide-binding site opener), which is able to activate Arf1 to enable WRC-dependent actin assembly. Furthermore, using labelled *E. coli*, we demonstrated that actin assembly by Arf6 also contributes towards efficient phagocytosis in THP-1 macrophages. Taken together, this study reveals a mechanism for Arf6-driven actin polymerization.

## 1. Introduction

The ADP-ribosylation factor (Arf) protein family is involved in a plethora of cellular functions, including endocytosis, vesicle trafficking, phagocytosis, and cytoskeleton remodeling [1]. The involvement of Arf GTPases in a wide array of cellular functions is partly attributed to their diverse localization within the cell. For instance, Arfs from class I (Arf1 and 3) and class II (Arf4 and 5) are predominantly localized around the Golgi apparatus and hence play a key role in vesicle, lipid, and organelle trafficking [2]. On the other hand, the only class III Arf (Arf6) is found exclusively at the plasma membrane and on incoming endosomes/macropinosomes and plays a vital role in endocytosis, exocytosis, receptor, and endosome recycling to the cell surface and has a clear role in cortical cytoskeleton rearrangement [3,4].

Like other GTPases, Arf GTPases are also under tight spatial and temporal regulation by their guanine nucleotide-exchange factors (GEFs) and GTPase-activating proteins (GAPs), which catalyze GTP binding and hydrolysis, respectively [5]. Arf GTPases can also coordinate with other GTPases, such as Rho and Rab, to further orchestrate precise functions within the cell [6,7].

Among the various tasks performed by Arf GTPases, we are interested in understanding how Arf GTPases regulate the actin cytoskeleton at the plasma membrane. Previously, we established that Rac1 co-ordinates with Arf1 at the plasma membrane to regulate actin rearrangement via the direct recruitment of the wave regulatory complex (WRC) [8] and this drives the formation of lamellipodia in cells [9,10]. The cooperative recruitment and activation of WRC at the membrane is not restricted to Arf1, as the related Arf5, and Arl1, a distant member of the Arf GTPase family, could also achieve similar activity. Furthermore, in contrast to other Arf family members, which directly bind and activate the WRC, Arf6 signaled to WRC indirectly by recruiting the Arf1 GEF ARNO to the plasma membrane [11,12,13]. Since Arf6 is implicated in numerous actin remodeling processes, we sought to find out whether Arf6 exclusively operates via this ARF1/ARNO pathway, or whether it can also directly activate WRC signaling.

## 2. Results

### 2.1. Constitutively Active Arf6 and Rac1 Cooperate in Triggering Actin Assembly In Vitro

**Figure 1:**
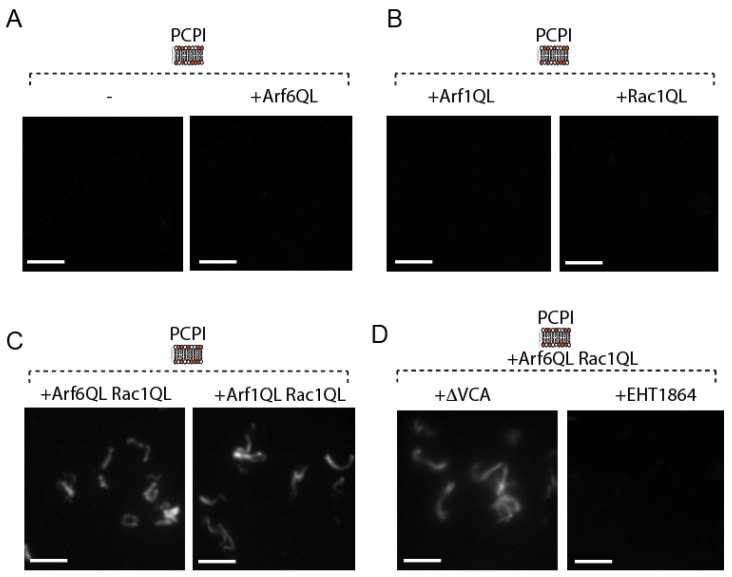
Fluorescence microscopy of rhodamine-actin assembly on the control, Arf6QL (**A**), Arf1QL, or Rac1QL (**B**) anchored PCPI membrane platforms in extract. (**C**) Fluorescence microscopy of rhodamine-actin assembly on Arf6QL:Rac1QL or Arf1QL:Rac1QL anchored PCPI membrane platforms in extract. (**D**) Rhodamine-actin assembly on Arf6QL: Rac1QL anchored PCPI membrane platforms in extract containing an inhibitor of Rac1 (EHT 1864) or N-WASP (n-waspΔvca) (scale bars: 10 µm).

In order to examine whether Arf6 could trigger actin assembly independently of ARNO, we looked at the ability of a constitutively active mutant of Arf6, i.e., Arf6Q67L, hereafter “Arf6QL”, to drive the formation of actin comets in porcine brain extract, as previously described [12,13,14]. We used porcine brain extract as we have shown previously that it lacks any detectable members of the ARNO family [12]. For this purpose, Arf6QL and Arf1QL, both alone and in combination with Rac1QL, were anchored to silica beads coated with a phospholipid bilayer composed of equal amounts of phosphatidylinositol and phosphatidylcholine (PC:PI). Control PC:PI lipid bilayers containing only Arf6QL, Arf1QL, or Rac1QL alone did not induce actin comet tail formation (Figure 1A,B). However, bilayers containing a combination of Arf6QL and Rac1QL were able to drive actin motility by forming actin comets similar to those achieved by Arf1QL and Rac1QL (Figure 1C). Inhibition of N-WASP-dependent activity by the addition of purified N-WASP ∆VCA had no effect on Arf6-driven actin assembly (Figure 1D), but actin motility was abolished by the addition of the Rac1 inhibitor EHT 1864, indicating that actin motility is Rac1 dependent (Figure 1D).

**Figure 2:**
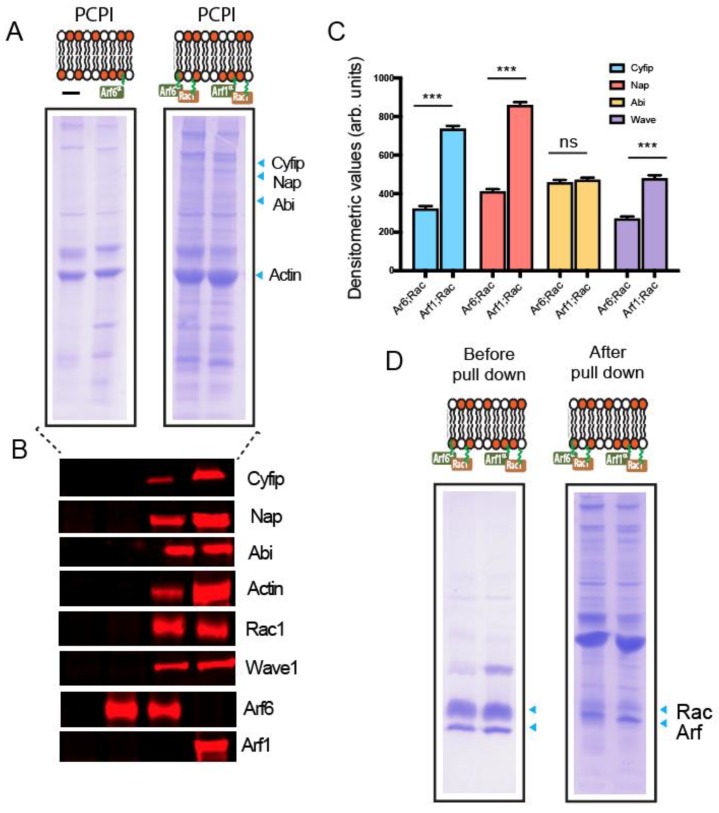
Coomassie blue staining depicting recruited protein from porcine brain extract on control (-), Arf6QL, Arf6QL; Rac1QL and Arf1QL; and Rac1QL anchored PCPI lipid bilayers (**A**). (**B**) Immunoblotting of samples from (**A**) with indicated antibodies. (**C**) Densitometric quantification of the wave-regulatory complex component bands recruited on Arf6QL; Rac1QL and Arf1QL; and Rac1QL anchored PCPI lipid bilayers as described in (**A**). (**D**) Coomassie blue staining depicting recruited proteins before and after incubating the Arf6QL; Rac1QL and Arf1QL; Rac1QL anchored PCPI lipid bilayers in brain extract. *** *p* < 0.001 (one-way ANOVA followed by a post hoc Dunnett comparison) relative to the control.

Rac1 is known to function upstream of WRC, but as we have previously shown [8], this requires a cooperating Arf GTPase. We therefore next sought to address whether Arf6 can cooperate with Rac1 to recruit WRC to the membrane. The anchored bilayers were incubated in porcine brain extract and the recruited proteins were subsequently analyzed by Coomassie Blue-stained SDS-PAGE, with their recruitment subsequently confirmed using immuno-blotting with the specific antibodies. As previously found [8], Rac1QL alone could not recruit any WRC components (Figure S1). However, as seen in Figure 2A,B, lipid bilayers containing both Arf6QL and Rac1QL recruited WRC components Cyfip, Nap1, WAVE1, and Abi1 similarly to Arf1QL:Rac1QL-containing bilayers. Densitometric quantification further revealed that Arf6QL:Rac1QL lipid bilayers had recruited approximately 50% less Cyfip, Nap1, and Wave than Arf1QL:Rac1QL bilayers (Figure 2C). Arf6 (WT) or a combination of Arf6WT: Rac1QL on PCPI monolayers failed to recruit WRC as shown in Figure S1. These differences in the WRC component recruitment are not due differences in the concentration of the GTPases present on the bilayers (Figure 2D). No recruited Arf1 was detected on theArf6QL:Rac1QL bilayers (using an antibody previously shown to detect porcine Arf1 [13]), suggesting that the WRC recruitment to these beads was achieved independently of Arf1. Taken together, these in vitro results suggest that Arf6 can recruit WRC independently of the ARNO-Arf1 signaling pathway. Lipid bilayers containing Arf6QL alone (control) did not recruit any detectable WRC components, thus this recruitment of WRC by Arf6 requires cooperation with Rac1.

### 2.2. Arf6 Regulates Phagocytosis in Differentiated THP-1 Human Macrophages

Various cellular functions are controlled by Arf family proteins, for example, they play a vital role in innate immunity by regulating phagocytosis. Both Arf1 and Arf6 are recognized as regulators of phagocytosis [15,16]. Previously, a number of studies [17,18,19] have indicated the involvement of both Arf6 and Arf1 in controlling phagocytosis by regulating actin assembly. Furthermore, we have previously shown [20] that Arf1 achieves this by directly mediating WRC actin polymerization, which was crucial for the phagocytosis of pathogenic *E. coli*. Thus, we next examined whether the Arf6- and Rac1-dependent WRC recruitment had any effect on phagocytosis.

**Figure 3:**
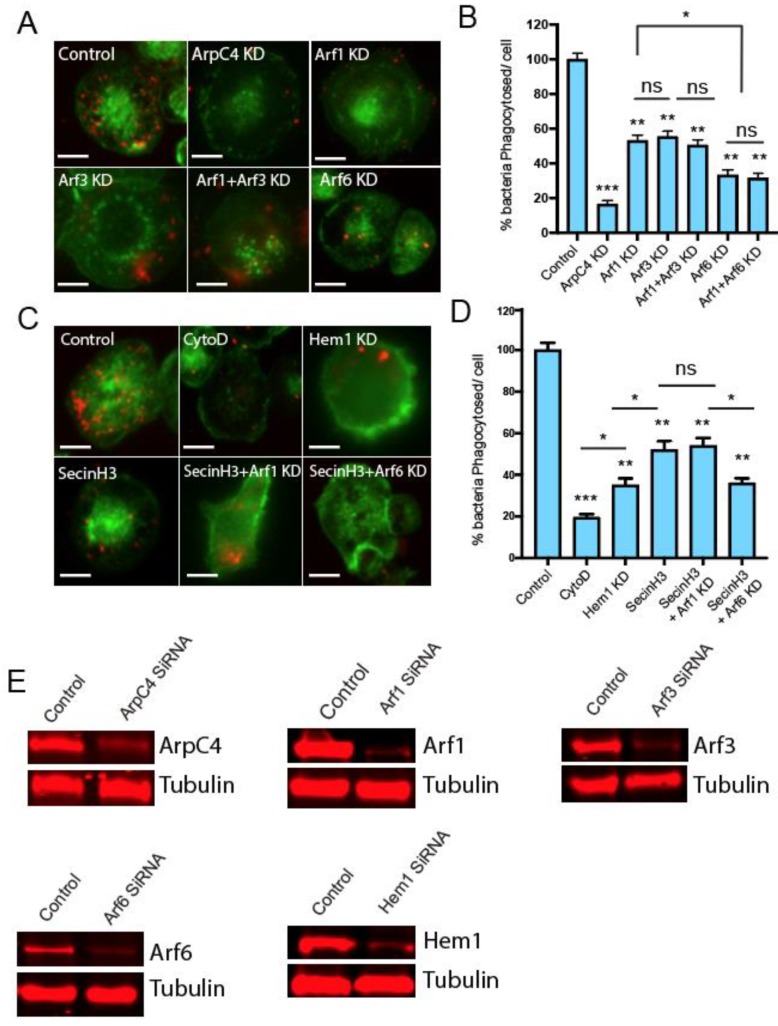
(**A**) Microscopy images depicting phagocytosis of labelled *E. coli* particles by PMA differentiated THP-1 macrophages (control) or upon silencing ArcC4, Arf1, Arf3, Arf1& Arf3, or Arf6 using siRNA. Internalized bacteria are shown in red while actin is stained using phalloidin (green). (**B**) Quantification of the phagocytosed *E. coli* by THP-1 macrophages as described in (**A**). Phagocytosis of phRodo-conjugated *E. coli* particles in THP-1 macrophages (control) or upon using actin inhibitor (CytoD), Arno inhibitor (SecinH3), or silencing of Arf1 or Arf6 in Arno-inhibited cells. Scale bar 10 µm (**D**). Represents quantification of the percentage of internalized bacteria under conditions as described for (**C**). (**E**) Immunoblot confirming the silencing of the mentioned proteins using SiRNA. Each bar represents the average of results from 3 separate experiments, and error bars represent SD, *** *p* < 0.001; ** *p* < 0.01; * *p*< 0.05; ns, not significant (one-way ANOVA followed by a post hoc Dunnett comparison) relative to the equivalent strain on WT THP-1 control cells. Lines indicate significance between pairs of conditions determined by Student’s *t* test.

For this purpose, we depleted Arf1, Arf6, or control ArpC4 (Arp2/3 component) in PMA differentiated THP-1 human macrophages using siRNA and measured the percentage of internalized phRodo-labelled *E. coli*. As shown in Figure 3A,B, the percentage of internalized bacteria decreased significantly (80%) in control ArpC4-depleted cells. The Arf1-depleted THP-1 macrophages had 45% less internalized bacteria, whereas Arf6 knockdown cells exhibited 65% less internalized bacteria. As Arf6 knockdown had a small, though significant, additional effect on the phagocytosis of labelled *E. coli* compared to Arf1 knockdown, this suggests that the role of Arf6 in promoting actin assembly is not simply to activate Arf1, and that parallel direct recruitment of WRC by Arf6 may also be important. Arf6 knockdown presumably inhibits both of these pathways. Consistent with this, Arf1 and Arf6 double knockdown exhibited phagocytosis comparable to Arf6 knockdown alone (Figure 3B and Figure S2A). The level of phagocytosis in Arf1–Arf6 double knockdown cells was comparable to that in Hem1 knockdown cells (Figure 3D), suggesting that the two Arf pathways are the major activators of WRC. As phagocytosis was reduced further in ArpC4 knockdown cells (Figure 3B), there must also be additional pathways operating to activate Arp2/3 independently of WRC.

It is reported that Arf3 can compensate for the loss of Arf1 [21,22] in regulating actin cytoskeleton dynamics. Hence, we decided to knockdown both Arf3 and Arf1 and then measure the phagocytosis of labelled *E. coli*. As shown in Figure 3C,D, the loss of Arf3 does not appear to further reduce phagocytosis, again suggesting that Arf6 is directly regulating actin assembly without the need for Arf1 or Arf3.

GEFs play an imperative role in activating small GTPases by stimulating GDP dissociation, which allows GTP binding. ARNO is reported to function as a GEF for both Arf1 and to a lesser extent Arf6 [23,24]; importantly, Arf6 activation can signal to Arf1 via recruiting and activating ARNO. Once activated, both Arf1 and Arf6 are capable of recruiting and activating more ARNO, leading to further activation of Arf1. We next examined whether the lower levels of phagocytosis observed in Arf6-depleted cells compared to Arf1 + Arf3-depleted cells were due to there being an Arf6-dependent but Arf1/3 + ARNO-independent mechanism.

For this purpose, the ARNO inhibitor SecinH3 was added to control, Arf1-depleted, or Arf6-depleted THP-1 differentiated cells and the ability to phagocytose labeled bacteria was assessed. As shown in Figure 3C,D, ARNO inhibition in control cells resulted in a decrease in phagocytosis similar to that observed upon Arf1 knockdown (Figure 3B). Furthermore, the depletion of Arf1 in SecinH3-treated cells had no significant additional effect to that of cells treated with SecinH3 alone, suggesting that both Arno and Arf1 drive phagocytosis via the same pathway. However, the depletion of Arf6 in SecinH3-treated cells resulted in a further 18% drop in phagocytosis, suggesting that Arf6 can also regulate phagocytosis independently of ARNO. To confirm whether this significant ARNO-independent activity of Arf6 was WRC dependent, we depleted Hem-1 (Nap1 equivalent in macrophages) in THP-1 cells. The Hem-1 knockdown resulted in levels of phagocytosis similar to those observed in Arf6-depleted cells (Figure 3B,D). Taken together, these results, and those above, indicate that Arf6 can signal to the actin cytoskeleton via the recruitment of WRC to regulate phagocytosis independently of ARNO and Arf1.

### 2.3. Arf6-Mediated Internalization of Salmonella is ARNO Dependent

To further confirm this, we examined *Salmonella* invasion in WT Hap, ΔArf6, and ΔNap1 Hap cells. *Salmonella* is a Gram-negative pathogen that uses its type-3 secretion system to force its entry into non-phagocytic cells by generating membrane ruffles that lead to macropinosome formation [25]. In order to generate these ruffles, *Salmonella* exploits WRC-mediated actin assembly. *Salmonella* activates Arf6 using host GEFs [12] and also generates PI(3,4,5)P_3_ in the plasma membrane. This leads to the activation of ARNO, which results in recruitment of Arf1 at the plasma membrane. Activated Arf1 and Rac1 can then trigger WRC-dependent membrane ruffles, leading to the internalization of *Salmonella* into the host cell.

We endeavored to use this system to examine whether the ARNO-independent ability of Arf6 to drive WRC-mediated actin polymerization also contributes to *Salmonella*’s invasion of non-phagocytic cells. Previously, all the work demonstrating the significance of Arf6 [12,13] in *Salmonella* invasion was based on siRNA-mediated silencing and drug-mediated inhibition.

**Figure 4:**
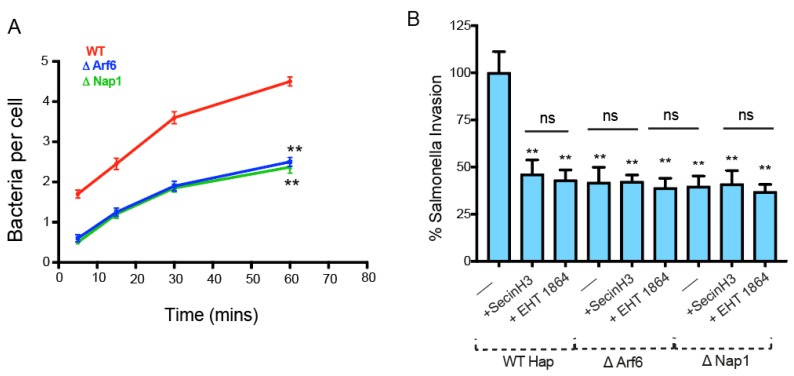
(**A**) Time course of *Salmonella* invasion in WT Haps, ΔArf6 (blue), or ΔNap1 (green) Hap cells with *Salmonella* bacteria carrying pM975 that express GFP inside pathogen-containing vacuoles. (**B**) Salmonella invasion in WT Haps, ΔArf6 Hap cells, or ΔNap1 Hap cells treated with Arno inhibitor (SecinH3) or Rac1 inhibitor (EHT 1864) after infecting *Salmonella* for 15 min. Each bar represents the average of the results from 3 separate experiments, and error bars represent SD. ** *p* < 0.01 (one-way ANOVA followed by a post hoc Dunnett comparison) relative to the control.

To more efficiently assess the role of Arf6, here, we used WT, ΔArf6, and ΔNap1 Hap1 cells, and performed a gentamycin protection assay measuring invasion at different times post infection (see methods). As can be seen in Figure 4A, in WT cells, the number of intracellular bacteria continued to increase until 60 min post-invasion. In both ΔArf6 and ΔNap1 cells, at all time points, there were significantly less internalized bacteria. Importantly, the inhibition of ARNO in WT cells impeded ī invasion to a similar level as that observed for Δ Arf6 cells. Subsequently, inhibiting ARNO (with secinH3) or Rac1 (using EHT 1864) did not further impede *Salmonella* invasion in ΔArf6 or ΔNap1 cells (Figure 4B), suggesting that in *Salmonella* entry, Arf6 acts exclusively via ARNO, signaling to WRC by recruiting ARf1 and that the Arf1/ARNO-independent pathway described above has a very small role in *Salmonella*’s invasion.

## 3. Discussion

Arf6 is the most predominant member of the Arf GTPase family that is extensively present at the plasma membrane. The role of Arf6 has long been established in regulating the actin cytoskeleton at the plasma membrane [26]. However, the molecular mechanism of how Arf6 directs actin polymerization is less well understood, majorly due to its diverse activities. Previously, our work has established that activation of Arf6, via receptor signaling, such as during EGF stimulation or indirectly by lipid modification by *Salmonella*, brings about actin polymerization at the leading edge of the cell by activating the Arf1 GEF ARNO. ARNO, upon activation, recruits Arf1 to the membrane, which can then co-ordinate with Rac1 to mediate actin polymerization via recruitment and activation of the WRC.

**Figure 5:**
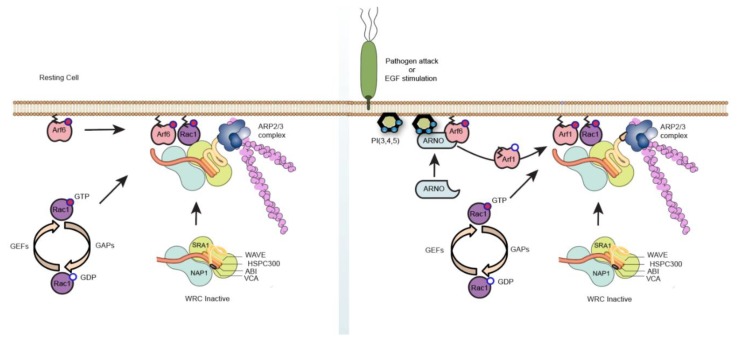
Schematic representation describing signaling to actin cytoskeleton by Arf6 GTPase. When the cell is in a resting state, Arf6 can mediate actin cytoskeleton rearrangement (via the wave regulatory complex) in co-operation with Rac1 to facilitate generic processes. This actin assembly is independent of ARNO and Arf1 interaction. On the contrary, when the cell is under an external stimulus, such as EGF, which results in acute PIP3 generation or during *Salmonella* entry, it switches to a more specific, stronger, short, and efficient means to bring about actin remodeling governed by ARNO/Arf1 recruitment, as described previously [12,13].

Here, by reconstituting actin assembly on lipid bilayers, we uncovered a potential direct role of Arf6 and demonstrated that it can assemble actin via the WRC, independently of ARNO. However, Arf6 has a poorer ability to drive WRC recruitment when compared to Arf1, as evident from the densitometric quantification of the recruited WRC components (Figure 2C). Hence, it is likely that Arf6 drives low levels of WRC activation, perhaps imitating the conditions in a resting unstimulated cell that would exhibit low-level actin remodeling for generic processes. When the cell is under an external stimulus, such as EGF, which results in acute PIP3 generation or during *Salmonella* entry [12,13], it switches to a more specific, stronger, short, and efficient means to bring about actin remodeling governed by Arf1 (Figure 5).

Both Arf1 and Arf6 are known for their role in phagocytosis [27,28]. Our results further illustrate that Arf6 by itself can contribute to phagocytosis by triggering WRC-mediated actin assembly without the involvement of Arf1/ARNO. Our results further provide a plausible explanation for the unexplained activation and localization of Arf6 at the tips of the pseudopods as previously reported by numerous studies [27,28,29]. Furthermore, these results are consistent with the reported role for Arf6 in Rac1 activation and lamellipodia formation [11,29].

Many pathogens have developed strategies to target Arf6 in order to facilitate their internationalization [1]. Whilst Arf6 can drive WRC activation independently of Arf1/ARNO as observed in vitro, the large reorganizations driven by *Salmonella* may well make use of the Arf6→ARNO→Arf1 pathway, which itself is amplified by positive feedback (as Arf1 can then recruit more ARNO), leading to a huge increase in WRC recruitment and activation required for this process [12,13]. The relatively low concentrated and scattered Arf6 in the cell alone is not sufficient, but the combination of Arf6 and PIP3 generation recruits ARNO, which activates Arf1, which in turn can recruit and activate more Arf1 at the plasma membrane [13]. This in turn results in far more efficient WRC recruitment and actin polymerization than that achieved by Arf6 alone. In a resting cell, Arf6 alone should not activate WRC unnecessarily, the requirement for a second downstream protein (Arf1 is required for acute short-lived but dramatic levels of actin turnover, driven by external stimuli, such as the enormous membrane ruffles generated by *Salmonella* during its invasion or when the cells are stimulated with epidermal growth factor (EGF)).

Our previous study [12] did not identify a direct role for Arf6 in actin remodeling, but it is worth noting that Arf6 loaded with the non-hydrolysable GTP analogue GTPγS was used to mimic active Arf6. In our original study, the loading of Arf6 with GTPγS was not quantified and thus may have been incomplete. In addition, the usage of GTPγS may not accurately recreate how Arf6 functions in a cell. The use of non-hydrolysable GTP analogues is reported to affect the activation state of other cellular proteins [30] and also GTPγS may not truly mimic the GTP-bound conformation of GTPase [31]. However, with the use of the constitutively active Arf6QL, we were able to uncover a potential direct role for Arf6 in regulating the actin cytoskeleton via the scar wave complex.

Unlike other Arfs, it seemed peculiar that Arf6 uniquely could not drive WRC activation; however, with better tools, we have now unraveled that Arf6 is directly capable of triggering actin polymerization independent of ARNO. Consistently, this ARNO-independent pathway of remodeling actin by Arf6 is distinct and may be utilized by the cell for more generic processes. Nonetheless, this study adds a new aspect of WRC control by defining how the Arf6 network further provides specificity in the regulation of WRC and highlights the elaborate spatiotemporal small GTPase control mechanisms that underlie actin polymerization specifically at the membrane.

## 4. Materials and Methods

### 4.1. Bacterial Strains

Salmonella SL1344 (gift from Jean Guard-Petter, Department of Agriculture, Athens, GA).

### 4.2. Antibodies

The following antibodies were purchased from Abcam (Rac1, ab33186; Arf6, ab81650; Arf1, ab58578; ArpC4, ab and tubulin, ab7291),Sigma (Abi1, A5106; actin, A2066; Cyfip, P0092; and Nap1, N3788) or were raised against recombinant peptides in rabbits by Diagnostics Scotland (WAVE1; amino acids 180–241).

### 4.3. Plasmids

The following plasmids were generated by Invitrogen Gateway methodology: pET20b-Arf6 encoding the Arf family *N*-myristoyltransferase site as previously described for Arf1, pET20b-Arf1, pET15b-Rac1 [8]. pM975-GFP from Wolf-Dietrich Hardt (EidgenössicheTechnische Hochschule, Zurich). GST- and His-tagged proteins were expressed in *Escherichia coli* Rosetta (Novagen, Merckmillipore, UK) at 18 °C overnight before affinity purification [12].

### 4.4. Cell Culture and Transfection

The human monocyte-like cell line THP1s (kind gift from Prof. Gordon Dougan) were cultured (37 °C 5% CO_2_) in RPMI-1640 supplemented with 10% heat-inactivated fetal calf serum (FCS), 200mg/mL–1 streptomycin, and 100 U mL–1penicillin. THP1s were differentiated into mature macrophage-like cells by stimulation with 100 ng/mL Phorbol 12-myristate 13-acae-tate (PMA) for 2 days and then cultured for an additional day without PMA before phagocytosis assays.

For RNAi, siRNA from Qiagen against Arf1 (Hs_Arf1_1 sequence ACGTGGAAACCGTGGAGTACA, Hs_ARF1_11 sequence AGGGAAGACCACGATCCTCTA), Arf3 (Hs_ARF3_3 sequence CAGGGCTGACTGGGTATTCTA, Hs_ARF3_5 sequence CACCTATATGACCAATCCCTA); ARF6 (Hs_ARF6_5 sequence CAACGTGGAGACGGTGACTTA, Hs_ARF6_7 sequence AAGACCAGTATAGTAAACTTA); ArpC4 (Hs_ARPC4_1 sequence CTGATAGGACCTTGATATATA, Hs_ARPC4_6 sequence CAGCATTAAAGCTGGCGCTTA); Hem1 (Hs_HEM1_1 sequence CAGGCATATACTAGTGTCTCA, Hs_HEM1_2 sequence TTCACTGAGATTATTCCTATA), or All Stars negative control siRNA (Qiagen) were combined for each individual gene (unless otherwise stated), and were introduced into differentiated THP1 cells with Oligofectamine transfection reagent (Invitrogen) according to the manufacturer’s instructions. The transfection mixture was replaced after 24 h with complete growth medium and cells cultured for an additional day before phagocytosis assay.

WT Hap1 (C631) and verified-knockout ΔArf6 (HZGHC003403c006), ΔNap1 (HZGHC003401c004) cell lines were purchased from Horizon Discovery. Hap1 cells were maintained in Iscove’s modified Dulbecco’s medium (IMDM) supplemented with 10% FBS and 100 U/mL penicillin-streptomycin.

### 4.5. Salmonella Invasion of Non-Phagocytic Host Cells

Wild-type *Salmonella enterica* serovar Typhimurium SL1344 were used to assay the invasion into non-phagocytic cells as previously described [12,13]. *Salmonella* encoding pM975 that expresses GFP via the SPI2 promoter when bacteria are within *Salmonella*-containing vacuoles (SCVs) [32] were used to infect WT HAP, Arf6, and Nap1 knockout cells (15 min unless otherwise stated), and then the number of fluorescent bacteria were counted per cell using fluorescence microscopy. When appropriate, WT HAP cells were pre-treated with the following small molecular inhibitors for 30 min prior to *Salmonella* infection, 10 µM EHT 1864 (Rac1), 5 µM CytoD (actin), and 25 µM SecinH3 (ARNO).

### 4.6. Phagocytosis Assay

Differentiated Human THP-1 cells were incubated at 37 °C with pHrodo *E. coli* bioparticles (ThermoFisher Scientific, P35361) for 60 min as per the manufacturer’s instructions, followed by fixing the cell using 4% paraformaldehyde (PFA) and actin was stained using AlexaFlour-488 phalloidin. Wherever indicated, cytochalasin (CytoD) or SecinH3 were added to the THP-1 cells 30 min prior to incubating the cells with pHrodo *E. coli* bioparticles. The amount of phagocytosis was assessed by counting the internalized bacteria (red) in a minimum of 50 cells per condition.

### 4.7. Actin-Based Motility and In Vitro Pull Downs

A 60-μL motility-mix (extract) was prepared on ice in the following order: 40 μL brain extract, 3 μL 20× energy mix (300 mM creatine phosphate, 40 mM MgCl2, 40 mM ATP), 3 μL G-actin/rhodamine actin, 6 μL 10× salt buffer (600 mM KCL, 200 mM 3-phosphoglycerate), 6 μL 50 mM BAPTA (Merck) and 1 μL 300 mM DTT (Merck), and, when appropriate, 1 μL 30 mM GTPγS (Roche). Actin-dependent motility assays were initiated by adding 0.1 vol phospholipids-coated beads to 10 μL motility mix, then 1μL was applied to a microscope slide and sealed under a glass coverslip with Vaseline:lanolin:paraffin (1∶1∶1), before viewing immediately under a fluorescence microscope (Leica DM IRBE) at Room Temperature. Digital images were captured (CCD camera, Hamamatsu) and analyzed (Volocity, Improvision), then figures assembled using Adobe Photoshop and Illustrator CS3.

The preparation of porcine brain extracts was as previously described [8,14]. Briefly, 40 fresh porcine brains were homogenized by 3 × 30 sec bursts of a Waring blender at 4 °C in an equal volume of extraction buffer (20 mM Hepes pH 7.4, 100 mM KCl 5 mM MgCl2, 0.5 mM EDTA, 1 mM EGTA with 0.5 mM ATP, 0.1 mM GTP, 1 mM DTT) supplemented with 10 μg/mL leupeptin, 10 μg/mL pepstatin, 10 μg/mL chymostatin, and Complete EDTA-free protease inhibitors (Roche). Homogenate was centrifuged (8000× *g*, 30 min, 4 °C) and the supernatant filtered through cheesecloth. Filtrate was clarified (12,000× *g*, 4 °C, 40 min), concentrated five-fold, and aliquots stored at −70 °C. Prior to use, thawed brain extract was clarified (100,000× *g*, 15 min).

For pull-down experiments, silica microspheres were coated with a bilayer composed of equal concentrations of phosphatidyl choline and phosphatidyl inositol (PC:PI). Indicated proteins (Arf6, Arf1, and Rac1) were anchored to these bilayers by incubating 15 μL of lipid-coated microspheres in 500 μL of HKS buffer containing approximately 20 μM of the protein to be anchored at room temperature for 1 h. The PC:PI bilayers were then washed by repeated (5×) low-speed centrifugation (1000× *g*) followed by resuspension in HKS buffer supplemented with 1 mM MgCl_2_ (HKSM). Micropsheres were finally resuspended in 15 μL of HKSM, and incubated with clarified porcine brain extract for 15min at room temperature. The bilayers were then washed 3 times with HKSM (as above), before the final resuspension in SDS-Urea, and recruited proteins were analyzed by SDS-PAGE, and where indicated by Western blotting.

### 4.8. Immunoblotting and Densitometric Quantification

Briefly, samples were run on a 4–12% Bis-Tris Protein Gel and then transferred to a nitrocellulose membrane using the iBlot2 (ThermoScientific). The membrane was then blocked using the Odyssey blocking buffer for 1 h and incubated overnight with primary antibody at 4 °C with rotation, washed with PBST, incubated with secondary antibody, washed with PBST, and imaged. All images were obtained using a LiCOR Odyssey imager and quantified with LiCOR software. For quantification, an area was drawn around the band of interest and the respective fluorescence was recorded and normalized to the fluorescence of the corresponding control band. Secondary antibodies were used at 1:5000 dilution and purchased from LiCOR Biosciences. The specific antibodies used were as follows: IRDye^®^ 800CW Goat anti Mouse IgG (925-32210), IRDye^®^ 680LT Goat anti Mouse IgG (925-68020), IRDye^®^ 800CW Goat anti Rabbit IgG (925-32211), and IRDye^®^ 680LT Goat anti Rabbit IgG (925-68021).

## 5. Conclusions

We have previously reported that Arf6 can promote actin assembly by triggering recruitment and activation of ARNO, which in turn activates Arf1 to cooperate with Rac1 in activating WRC. Here, we have shown that in addition to this indirect pathway, Arf6 is able to directly recruit and activate WRC. This direct activity also requires cooperating Rac1, but is independent of the ARNO/Arf1 pathway. Both Arf6 pathways operate in THP-1 macrophages to drive the actin rearrangements necessary for phagocytosis. This work thus represents a new aspect of WRC control by a complex network of cooperating GTPases.

## Supplementary Materials

Supplementary materials can be found at https://www.mdpi.com/1422-0067/21/7/2457/s1. Figure S1. Coomassie blue staining depicting recruited protein from porcine brain extract on control (-) and Rac1QL anchored PCPI lipid bilayers(A). (B) Immunoblotting of samples from (A) with indicated antibodies. (C) Coomassie blue staining depicting recruited protein from porcine brain extract on control Arf6WT (alone), and a combination of Arf6WT; Rac1QL anchored PCPI lipid bilayers. (D) Immunoblotting of samples from (C) depicting recruited proteins as indicated antibodies. Figure S2. (A) Microscopy images depicting phagocytosis of labelled E. coli particles by PMA differentiated THP-1 macrophages (control) or upon silencing Arf1 + Arf6 using siRNA. Internalized bacteria are shown in red while actin is stained using phalloidin (green). (B) Immunoblot confirming the silencing of the mentioned proteins using SiRNA. (C) Summarized Densitometric quantification depicting the efficiency of the silenced proteins as determined and normalized to control cell.
