# Supplementary material for: Arf6 Can Trigger Wave Regulatory Complex-Dependent Actin Assembly Independent of Arno
Source: Int J Mol Sci. 2020 Apr 2;21(7):2457. doi: 10.3390/ijms21072457 (PMC7177560; doi:10.3390/ijms21072457)
Supplement: Supplementary file 1 [file ijms-21-02457-s001.pdf]

Supplementary

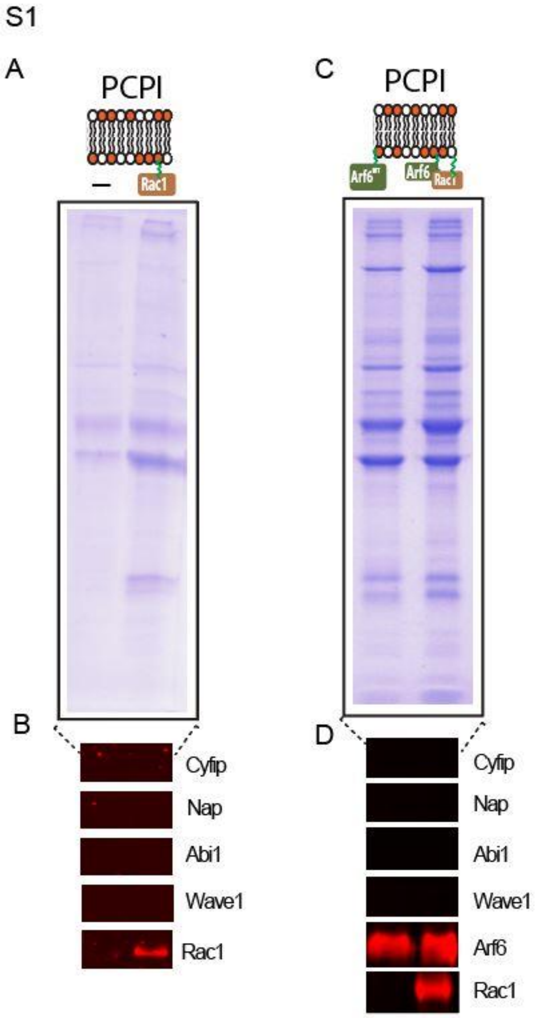

Figure S1. Coomassie blue staining depicting recruited protein from porcine brain extract on control (-) and Rac1QL anchored PCPI lipid bilayers(**A**). (**B**) Immunoblotting of samples from (**A**) with indicated antibodies. (**C**) Coomassie blue staining depicting recruited protein from porcine brain extract on control Arf6WT (alone), and a combination of Arf6WT; Rac1QL anchored PCPI lipid bilayers. (**D**) Immunoblotting of samples from (**C**) depicting recruited proteins as indicated antibodies.

S2

A B

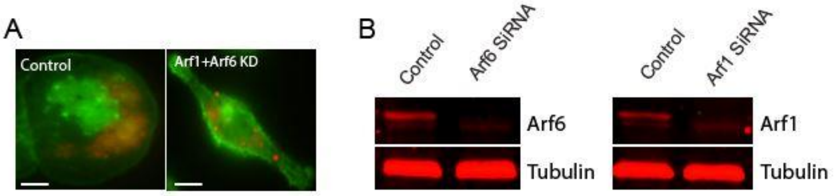

C

Targeted Protein	Knockdown Efficiency
ArpC4	72%
Arf1	69%
Arf3	59%
Arf1+ Arf3	Arf1 (70%); Arf3 (55%)
Arf6	74%
Arf1+ Arf6	Arf1 (69%); Arf6 (71%)
Hem1	70%

Figure S2. (**A**) Microscopy images depicting phagocytosis of labelled *E.coli* particles by PMA differentiated THP-1 macrophages (control) or upon silencing Arf1+Arf6 using siRNA. Internalized bacteria are shown in red while actin is stained using phalloidin (green). (**B**) Immunoblot confirming the silencing of the mentioned proteins using SiRNA. (**C**) Summarized Densitometric quantification depicting the efficiency of the silenced proteins as determined and normalized to control cell.
